# Laienreanimationstraining in Österreich: Eine Übersicht und Annäherung

**DOI:** 10.1007/s00508-024-02331-7

**Published:** 2024-02-01

**Authors:** Christoph Veigl, Simon Orlob, Thomas Kloimstein, Benedikt Schnaubelt, Mario Krammel, Markus Draxl, Lukas Feurhuber, Johannes Wittig, Joachim Schlieber, Sebastian Schnaubelt

**Affiliations:** 1 Universitätsklinik für Notfallmedizin, Medizinische Universität Wien, Währinger Gürtel 18–20, 1090 Wien, Österreich; 2 PULS – Verein zur Bekämpfung des plötzlichen Herztodes, Wien, Österreich; 3 Österreichischer Rat für Wiederbelebung (ARC), Graz, Österreich; 4 Universitätsklinik für Anästhesiologie und Intensivmedizin, Medizinische Universität Graz, Graz, Österreich; 5 Drück Mich! Arbeitsgemeinschaft für Notfallmedizin, Graz, Österreich; 6 Ordensklinikum Linz Elisabethinen, Linz, Österreich; 7 SINUS – Interdisziplinäre Notfallinitiative Linz, Linz, Österreich; 8 Zurück ins Leben, Horn, Österreich; 9 Berufsrettung Wien (MA 70), Wien, Österreich; 10 Medizinische Universität Innsbruck, Innsbruck, Österreich; 11 IGNI – Interessengemeinschaft Notfallmedizin Innsbruck, Innsbruck, Österreich; 12 Karl Landsteiner Privatuniversität für Gesundheitswissenschaften, Krems an der Donau, Österreich; 13 emerKREMSy – studentischer Verband für Notfallmedizin Krems, Krems an der Donau, Österreich; 14 Research Center for Emergency Medicine, Universityhospital Aarhus, Aarhus, Dänemark; 15 Randers Regional Hospital, Randers, Dänemark; 16 Abteilung für Anästhesiologie und Intensivmedizin, Unfallkrankenhaus Salzburg, Salzburg, Österreich

**Keywords:** Kardiopulmonale Reanimation, Herzstillstand, Plötzlicher Herztod, Awareness, Basic life support, Cardiopulmonary resuscitation, Out-of-hospital cardiac arrest, Sudden cardiac death, Awareness, Basic life support

## Abstract

Das frühzeitige Eingreifen von Laien bei einem außerklinischen Herzstillstand verbessert die Überlebenswahrscheinlichkeit und das neurologische Outcome erheblich. In Österreich gibt es einige Organisationen und Initiativen, die durch Reanimationsschulungen für Laien einerseits- und Awarenessbildung für den plötzlichen Herztod andererseits versuchen, die aktuell niedrige Überlebenswahrscheinlichkeit des plötzlichen Herztodes zu steigern. Um eine Übersicht über die verschiedenen Organisationen zu erhalten, wurden Fragebögen versandt. Fünfzehn von 26 Organisationen (58 %) beantworteten den Fragebogen. Die geografische Verteilung der Organisationen zwischen ländlichem und städtischem Gebiet wurde anhand einer Karte dargestellt. Ein großer Teil der Organisationen ist in Universitätsstädten aktiv. Dies führt zu einer unterschiedlichen Erreichbarkeit von Personen im ländlichen und urbanen Raum. Die Schulung von Laien in der Reanimation ist größtenteils von ehrenamtlichem Engagement abhängig. Die verwendete Zeit für das praktische Üben der Herzdruckmassage in Reanimationskursen variiert zwischen 25 und 90 % der gesamten Zeit eines Kurses. Weiters konnten Gründe für ein fehlendes wissenschaftliches Engagement exploriert werden. Durch die Vernetzung der verschiedenen Organisationen und Initiativen sollen in Zukunft noch mehr Laien geschult werden als bisher, um dadurch die Überlebenswahrscheinlichkeit des außerklinischen Kreislaufstillstands in Österreich zu steigern. Eine entsprechende Unterstützung durch die Politik und öffentliche Instanzen sollte selbstverständlich sein bzw. werden.

**Zusatzmaterial online:**

Zusätzliche Informationen sind in der Online-Version dieses Artikels (10.1007/s00508-024-02331-7) enthalten.

## Abstract (en)

Early interventions of laypersons can improve the survival and neurological outcome in patients with out-of-hospital cardiac arrest. There are several organizations in Austria which train lay people in basic life support and raise awareness for sudden cardiac death. To obtain an overview of the various initiatives, a questionnaire was sent to 26 organizations, and 15 of the organizations (58%) replied. The geographical distribution of the organizations between rural and urban areas was illustrated in a map. Most of them are situated in a university city, resulting in accessibility disparities for individuals in urban and rural settings. Layperson resuscitation education in Austria is largely dependent on the individual commitments of volunteers. The time spent practicing chest compressions in resuscitation courses ranges from 25% to 90% of the total course time. Furthermore, reasons for a lack of scientific endeavours could be identified, and solutions are suggested. Through better networking between organizations and initiatives, more laypersons could be trained in the future, which would lead to improved survival chances for persons suffering from out-of-hospital cardiac arrest in Austria. Appropriate support by political bodies and public authorities is and will remain a key element.

## Hintergrund

Die Überlebenswahrscheinlichkeit eines plötzlichen Herztodes bleibt weltweit trotz zahlreicher Weiterentwicklungen in der Notfall- und Intensivmedizin in den letzten Jahrzehnten weiterhin niedrig [1, 2]. Viele der Fälle finden außerhalb des Krankenhauses statt – daher spielen Laien eine entscheidende Rolle in der in der Überlebenskette [3], die initial durch den Österreicher Peter Safar entscheidend geprägt wurde [4]: Die Minuten bis zum Eintreffen des professionellen Rettungsdienstes müssen durch eine effektive Basisreanimation der Umstehenden überbrückt werden. Die ersten drei Schritte der Überlebenskette, nämlich das rasche Erkennen von Symptomen schon vor dem Kreislaufstillstand, das Rufen von Hilfe, und die frühe Herzdruckmassage und Defibrillation werden den Basismaßnahmen der Wiederbelebung zugerechnet [3, 5]. Schnell und richtig angewandt vergrößern diese die Chance einen außerklinischen Kreislaufstillstand („out-of-hospital cardiac arrest“ [OHCA]) mit gutem neurologischem Outcome zu überleben erheblich [6]. Trotz der enormen Wichtigkeit dieser lebensrettenden Sofortmaßnahmen gibt es bei Laien große Wissenslücken [7–9]. In der Vergangenheit wurden bereits Hindernisse identifiziert, die Laien von der Teilnahme an Reanimationsschulungen abhalten – beispielsweise ein höheres Alter, ein niedrigerer sozioökonomischer- und Bildungsstatus, Sprachbarrieren oder die Zugehörigkeit zu Randgruppen [10]. Einen wichtigen Beitrag um diese Lücken zu schließen und Überlebensraten zu steigern, spielen nationale und lokale Initiativen mit Bezug zur Laienreanimation [11]. Diese machen auf die Gefahren des plötzlichen Herztodes aufmerksam und lehren Basiswiederbelebungsmaßnahmen. Mit gezielten und speziell angepassten Reanimationstrainings können so Barrieren in verschiedenen Bevölkerungsgruppen abgebaut und so auch jene Gruppen erreicht werden, in welchen bisher etwaige Wissenslücken aufgrund der beschriebenen Hindernissen besonders groß waren [12]. So spielen derartige Organisationen und Initiativen auch im kürzlich vorgestellten „chainmail of survival“ eine zentrale Rolle [13], und die Wichtigkeit von funktionierenden First-Responder-Systemen, die angeleitete Telefonreanimation, und das frühzeitige Schulen von Kindern werden auch in den aktuellen Leitlinien des Europäischen Rates für Wiederbelebung (ERC) betont [3].

In Österreich existieren einige Organisationen, Vereine und Initiativen *(im Nachfolgenden nur noch „Organisationen“ genannt),* die auf das Thema des plötzlichen Herztodes aufmerksam machen und die Bevölkerung in Wiederbelebungsmaßnahmen trainieren. Teilweise werden diese Bemühungen auch wissenschaftlich begleitet und evaluiert, und es konnten bereits positive „Durchdringungseffekte“ in Österreich gezeigt werden: Beispielsweise wurde durch ein Kooperationsprojekt zwischen dem Verein PULS und der Wiener Polizei die Überlebensrate nach OHCA gesteigert [14, 15].

In diesem Artikel soll eine Übersicht über diese verschiedenen Organisationen und ihre Projekte gegeben werden, um die Vernetzung und Zusammenarbeit zu fördern und die – österreichweit größtenteils ehrenamtlich – geleistete Arbeit sichtbar zu machen.

## Materialien und Methoden

Nationale und regionale Organisationen mit Bezug zur Laienreanimation und entsprechender Awarenessbildung in Österreich wurden durch Experten identifiziert und zur Teilnahme an einer Umfrage eingeladen. Zusätzlich wurden alle in Österreich tätigen Rettungsdienste befragt, welche laut ihrer Website Erste-Hilfe-Kurse oder Ähnliches anbieten. Insgesamt wurde somit ein Fragebogen im Juni 2023 an 26 unterschiedliche Organisationen verschickt. Es konnten ausschließlich die eingeladenen Organisationen an der Umfrage teilnehmen.

Der Fragebogen *(Online-Anhang)* wurde durch ein Team aus Experten erstellt und beinhaltet neben allgemeinen Informationen zu den Organisationen auch Angaben zu den verschiedenen Kursformaten, Awarenessbildungsmaßnahmen und einer etwaigen wissenschaftlichen Tätigkeit. Die teilnehmenden Organisationen stimmten vor der Beantwortung der Veröffentlichung ihrer Daten schriftlich zu.

## Ergebnisse

**Abb. 1:**
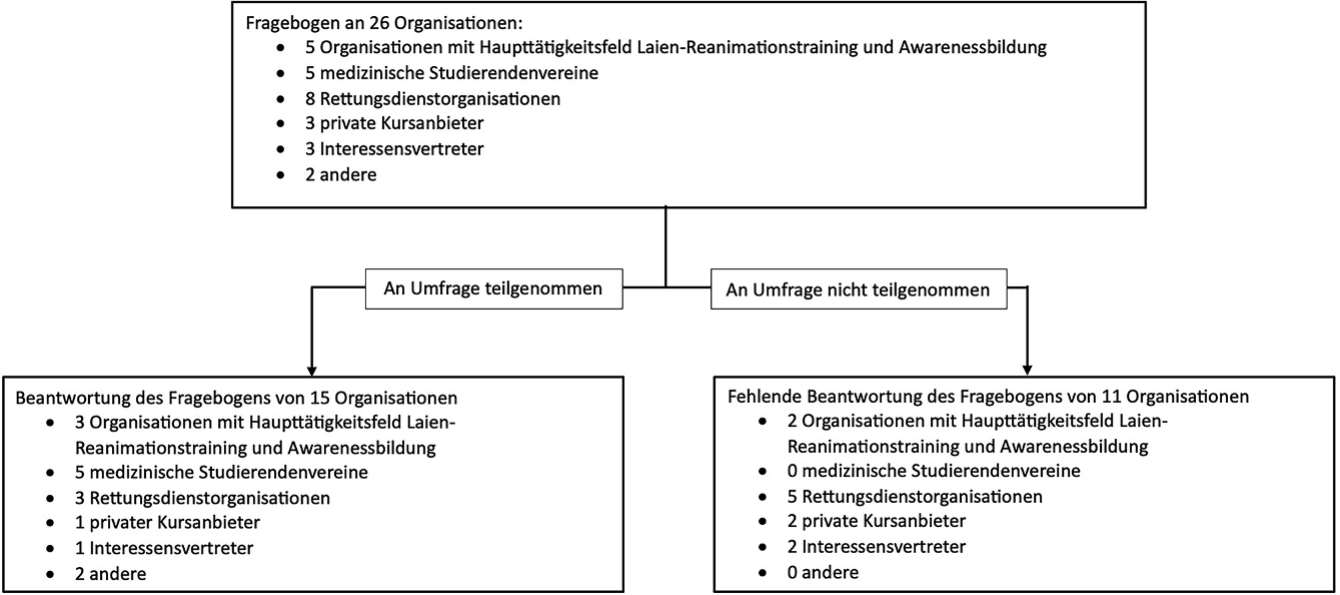
Übersicht über die kontaktierten Organisationen

Insgesamt beantworteten 15 von 26 Organisationen (58 %) den Fragebogen (Abb. 1). Von den elf fehlenden Organisationen sind fünf dem Rettungsdienst zuzuordnen *(Anm.: Die Berufsrettung Wien wurde nicht kontaktiert, da diese keine Laienausbildung durchführt)*.

## Geografische Verteilung und Tätigkeitsfelder

**Abb. 2:**
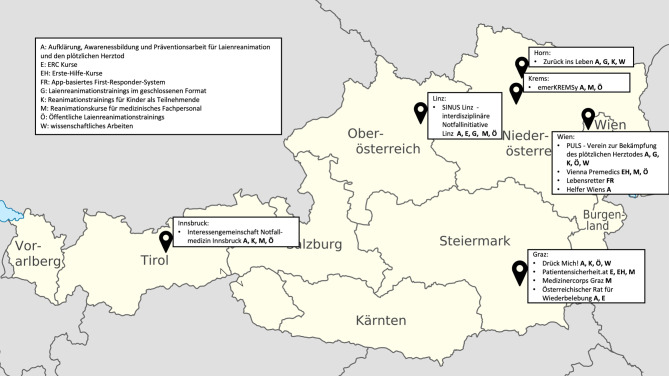
Österreichkarte mit den Organisationen mit Bezug zur Laienreanimation sowie deren jeweilige Tätigkeitsbereiche. Auf Rettungsdienstorganisationen wurde bei dieser Abbildung verzichtet. (*Adaptiert durch C. Veigl nach* [16])

Abb. 2 zeigt die Standorte der inkludierten Organisationen incl. der jeweiligen Tätigkeitsbereiche im Kontext der Reanimation. Aufgrund der geringen Rücklaufrate bei den Rettungsdienstorganisationen und der meist strikten Bundeslandaufteilung der jeweiligen Landesverbände wurde auf diese bei der Abbildung verzichtet.

Bei sieben von 15 der teilnehmenden Organisationen (47 %) waren die Awarenessbildung für die Laienreanimation und den plötzlichen Herztod sowie das Lehren der Laienreanimation die Hauptziele bei der Gründung. Aktuell bieten 10 der 15 Organisationen (67 %) Reanimationstrainings für Laien an. Die Reanimationstrainings reichen von Kurztrainings im Rahmen von öffentlichen Veranstaltungen oder Aktionstagen bis hin zu mehrstündigen Kursen im geschlossenen Format.

## Kursformate, Vortragende und Kursevaluierung

Zwei Organisationen (20 %) bieten Laientrainings ausschließlich im geschlossenen Format im Rahmen eines Kurses an, drei Organisationen (33 %) ausschließlich im Rahmen von öffentlichen Veranstaltungen oder Aktionstagen, und fünf Organisationen (50 %) beides. Insgesamt werden also von sieben Organisationen (47 %) Reanimationskurse im geschlossenen Format im Rahmen eines Kurses angeboten. Sieben der 10 Organisationen (70 %), welche Laienreanimationstrainings anbieten, tun dies komplett kostenlos, und die weitern drei (30 %) verlangen zumindest bei bestimmten Kursformaten eine Teilnahmegebühr. Die Kursdauer variiert zwischen 90 min und bis zu vier Stunden. Generell schwankt die Zeit, die innerhalb eines Kurses für das praktische Üben der Reanimation verwendet wird, zwischen ca. 25 und 90 %. Die Kurse werden in verschiedenen Räumlichkeiten angeboten, wobei sie sowohl in Räumlichkeiten der Organisationen selbst (71 %), in Firmen (57 %), Schulen (42 %) oder öffentlichen Gebäuden (42 %) stattfinden. Bei Rettungsorganisationen tragen bei Kursen Erste-Hilfe-Lehrbeauftragte und medizinisches Fachpersonal vor, ansonsten ist die Mindestqualifikation der TrainerInnen für die Kurse nicht einheitlich und reicht von internen Einschulungen ohne vorherige Mindestqualifikation über allgemeines medizinisches Fachpersonal bis hin zu InstruktorInnen von Kursen des ERC. Die Vortragenden sind jeweils bei drei Organisationen (43 %) entweder gemischt (hauptamtlich und ehrenamtlich) oder rein ehrenamtlich tätig. Bei der einen verbleibenden Organisation (14 %) trägt nur hauptamtliches Personal vor. Bei sechs von sieben Organisationen (86 %) wird die Qualität des Reanimationskurses evaluiert: Die häufigste Form (83 %) sind Fragebögen. Zusätzlich werden noch Tests vor und nach den Kursen zur Überprüfung des Fortschritts der Teilnehmenden (17 %) sowie eine interne Evaluierung der Kursqualität (17 %) eingesetzt. Um die Qualität der Vortragenden aufrechtzuhalten, absolvieren die Vortragenden bei zwei Organisationen (33 %) regelmäßig verpflichtende Fortbildungen, bei drei Organisationen (50 %) regelmäßige freiwillige Fortbildungen, und bei vier (67 %) findet eine regelmäßige Evaluierung der Vortragenden statt.

## Erste-Hilfe-Kurse

Allgemeine Erste-Hilfe-Kurse, bei denen die Reanimation zumindest in einem Teilaspekt behandelt wird, werden von vier der 15 Organisationen (27 %) angeboten, wovon zwei Organisationen dem Rettungsdienst zuordenbar sind.

## Reanimationskurse für Kinder

Ein spezieller Reanimationskurs für Kinder als Teilnehmende wird von acht der 15 Organisationen (53 %) angeboten. Die Kurse dauern zwischen ein und zwei Stunden und finden bei sechs Organisationen (88 %) in Schulen statt, bei zwei Organisationen (25 %) in eigenen Räumlichkeiten, und bei einer Organisation (12 %) in anderen öffentlichen Gebäuden. Bei diesen Kursen werden mindestens 50 bis 80 % der Zeit für das praktische Üben der Reanimation verwendet. Die Vortragenden bei Kursen für Kinder sind bei einer Organisation (12 %) nur hauptamtliches Personal, bei drei (38 %) ehrenamtlich und bei den restlichen vier Organisationen (50 %) gemischt. Eine Organisation (12 %) verlangt eine spezielle verpflichtende Schulung für die Vortragenden, und bei jeweils einer Organisation findet die Evaluierung der Kurse mittels Fragebogen oder direkter Evaluation statt.

## Aufklärung, Awarenessbildung und Präventionsarbeit für Laienreanimation und den plötzlichen Herztod

Eine verbesserte öffentliche Wahrnehmung für die Laienreanimation und den plötzlichen Herztod durch Awarenessbildung wird durch 13 von 15 teilnehmenden Organisationen (87 %) angestrebt. Dafür werden verschiedene Möglichkeiten genutzt, beispielsweise Social-Media-Kanäle (von elf Organisationen [85 %], und damit am häufigsten aller Methoden). An Aktionstagen bezüglich des plötzlichen Herztodes beteiligen sich neun der 13 teilnehmenden Organisationen (69 %) durch öffentliche Reanimationstrainings oder Aufklärungskampagnen. Ein Beispiel für diese Aktionstage ist der World Restart a Heart Day. Dieser wurde 2018 erstmalig unter dem Motto „All citizens of the world can save a life“ abgehalten und findet seitdem jährlich mit einem neuen Motto statt [17, 18]. Sechs der 13 Organisationen (46 %), die Aufklärungsarbeit leisten, sind regelmäßig mit Ständen an öffentlichen Veranstaltungen vertreten. Bei diesen Ständen können in kurzer Zeit zahlreiche Laien erreicht werden, bei denen dann ein mögliches Vorwissen aufgefrischt, Aufmerksamkeit für das Thema generiert, oder ein Anstoß gegeben wird, einen längeren Reanimationskurs zu besuchen. Wichtig ist jedoch, dass solche Kurzschulungen kein mehrstündiges Reanimationstraining ersetzen können. Forderungen und Vorschläge an die Politik werden von sechs der 13 Organisationen (46 %) formuliert.

## Wissenschaftliches Arbeiten

Die Erstellung bzw. Mitarbeit an Studien mit Bezug zur Laienreanimation findet bei vier von 15 Organisationen (27 %) statt. Als Gründe für die fehlende wissenschaftliche Arbeit in diesem Bereich werden vor allem eine mangelnde Finanzierung, fehlende zeitliche Valenzen oder fehlenden Kontakte zu wissenschaftlichen Partnern angegeben. Durch tiefere Einblicke in die Arbeit von wissenschaftlichen Partnern kann aber die Grundlage für eine erfolgreiche Zusammenarbeit entstehen – In Zukunft sollten Forschung und die praktische Ausbildung von Laien präferenziell nicht getrennt, sondern in enger Vernetzung erfolgen.

## Finanzierung

Ausschließlich eine (7 %) der teilnehmenden Organisationen arbeitet gewinnorientiert. Die Finanzierung der Organisationen ist vielfältig und reicht von Förderungen von Gemeinde, Land und Bund über Kurskosten, Mitgliedsbeiträge und Spenden bis hin zu Förderungen der Hochschulschaft. In 13 von 15 Organisationen (87 %) kann man sich ehrenamtlich engagieren, und nur vier von 15 (27 %) haben hauptamtliches Personal angestellt, das finanziert werden muss.

## Diskussion

In Österreich existiert eine größtenteils ehrenamtlich engagierte Landschaft der expliziten Laienreanimationsschulung, und diese ist fast ausschließlich regional organisiert. Wie in Abb. 2 ersichtlich, gibt es in einigen Städten mindestens eine Organisation, welche öffentliche Reanimationstrainings für Laien anbietet. Jedoch sind die entsprechenden Organisationen vorrangig in Universitätsstädten tätig; dies ist am ehesten durch die Verbindungen der dort tätigen „key player“ mit einer Universität erklärbar, und eine zukunftsgerichtete, praxis- und gesellschaftsnahe, universitär-medizinische Ausbildung [19] kann grundsätzlich von solchen Kollaborationen profitieren. Jedoch kann es dadurch auch deutlich schwieriger sein, die Bevölkerung im ländlichen Bereich zu erreichen, und vor dem Hintergrund langer Eintreffzeiten des Rettungsdienstes im ländlichen Bereich ergibt sich ein gefährlicher Mix für die Betroffenen: Es dauert nicht nur länger, bis professionelle Hilfe eintrifft, sondern es könnte durch eine geringere „Abdeckung“ hinsichtlich Awarenessbildung und Laienschulungen auch weniger wahrscheinlich sein, dass eine suffiziente Laienreanimation zur Zeitüberbrückung stattfindet. Fehlende niederschwellige und kostenlose Trainingsmöglichkeiten für Laien durch (fehlende) lokale Organisationen könnten somit ein Grund für die geringere Überlebenswahrscheinlichkeit des OHCAs im ländlichen Raum sein [20]. Ebenfalls anhand der Karte ersichtlich sind ein starkes Ost-West-Gefälle und die fehlende Abdeckung weiter Gebiete im Westen. In Zukunft sollten durch die Politik Anstrengungen entstehen die genannten Lücken zu schließen und für Organisationen Anreize und Unterstützungen zu schaffen, bisher suboptimal betreute Gebiete abzudecken.

### Vortragende und Train-the-trainer-Konzept

Unsere Umfrage zeigt, dass die Ausbildung von Laien stark von kostengünstigem ehrenamtlichen Engagement abhängig ist. Außerdem gibt es offenbar keine einheitliche Mindestqualifikation für die eingesetzten Vortragenden. In allen teilnehmenden Organisationen sind ehrenamtliche Mitarbeiter als Vortragende tätig, und in 70 % der Fälle erfolgt die Laienausbildung sogar ausschließlich durch Freiwillige. In den letzten Jahren nimmt die ehrenamtliche Arbeit in Österreich jedoch ab [21]. Folglich sollten die Organisationen Strategien entwickeln, um ehrenamtliche Mitarbeiterinnen und Mitarbeiter zu gewinnen und diese auch über längere Zeiträume für eine aktive Mitarbeit zu motivieren. Beispielsweise könnte eine fundierte Aus- und Weiterbildung im Sinne eines „Train-the-Trainer“-Konzepts für mehr Fachkenntnis und ein besseres subjektives Gefühl während des Abhaltens von Kursen sorgen [22]. Im Rahmen eines solchen Konzepts sollten die Organisationen ihre Trainer und Vortragenden in der primären Aus- und der nachfolgenden Weiterbildung begleiten. Beispielsweise können auf eine initiale „hausinterne“ Ausbildung regelmäßige Fortbildungen folgen (z. B. medizinische Fachvorträge, Kongresse oder pädagogische Weiterbildungen). Bei der Planung muss von den Organisationen darauf geachtet werden, dass auch ehrenamtliches Personal berufsbegleitend teilnehmen kann.

### Ablauf eines Reanimationstrainings

Generell schwankt die Zeit, die für das praktische Üben der Reanimation in den unterschiedlichen Kursen eingeplant ist, von Organisation zu Organisation sehr stark. Jin Hyuck Lee et al. konnten nachweisen, dass es einen signifikanten Unterschied im Trainingseffekt von Basiswiederbelebungsmaßnahmen durch Laien gibt, abhängig davon, ob ein Kurs 80 oder 120 min dauert [23]. Um die Kursqualität österreichweit einheitlich zu gestalten, sollten abhängig von der Kursdauer und der Anzahl an TeilnehmerInnen jedoch mindestens 90 min für das praktische Üben der Basiswiederbelebung eingeplant werden, und die Vortragenden sowohl das erforderliche medizinische als auch das pädagogische Wissen vorweisen können. Als selbstverständlich sollte gelten, dass jede/r Teilnehmende/r zumindest so lange praktisch an einer Puppe übt, bis ein suffizientes Ingangsetzen der Rettungskette, ein korrektes Feststellen des Kreislaufstillstandes, und eine hochqualitative Herzdruckmassage beobachtet werden konnten. Ein manchmal übliches kurzes Vorzeigen der Reanimation und Fragen in die Runde „Wer möchte mal probieren?“ ist definitiv nicht ausreichend und darf – z. B. in Führerschein-Erste-Hilfe-Kursen – nicht den Eindruck vermitteln, große Teile der Bevölkerung in Reanimation geschult zu haben. Entweder muss bei generellen Erste-Hilfe-Kursen die aufgewendete Zeit für das Lehren und Üben der Reanimation ausgedehnt werden [23], oder es müssen zusätzliche Anreize für die Allgemeinbevölkerung geschaffen werden, regelmäßig spezielle Reanimationskurse zu besuchen.

### Neue Ausbildungskonzepte und Kooperationen zwischen Organisationen

Um neue fundierte Ausbildungskonzepte für Laien zu etablieren, ist es notwendig, diese im Rahmen von Studien mit einheitlichen Standards zu überprüfen [24]. Dazu muss in Zukunft eine bessere Zusammenarbeit zwischen im Bereich Laienreanimation tätigen Organisationen und wissenschaftlichen Partnern stattfinden, und es braucht hierfür auch eine entsprechende (öffentliche) Finanzierung. Dadurch kann die Ausbildung für Laien weiter ausgedehnt werden, um die niedrige Überlebenswahrscheinlichkeit und das oftmals schlechte neurologische Outcome zu verbessern. Zusätzlich zur Rettung von Menschenleben spart dies gesundheitsökonomisch weitere Behandlungs- und Betreuungskosten ein [25, 26]. Um die Jüngsten der Gesellschaft zu erreichen, sollte ein verpflichtendes, zumindest jährliches Reanimationstraining in Schulen angeboten werden [27]. Dafür sind nicht nur die Finanzierung durch den Staat, sondern auch ein fundiertes Ausbildungskonzept für die Vortragenden notwendig. In einer kürzlich erschienenen *Lancet Commission* zum Thema des plötzlichen Herztodes wurden auch zahlreiche weitere solcher Forderungen an die Politik gerichtet [28].

Zukünftige Kooperationen zwischen den Organisationen könnten genutzt werden, um Trainingsmöglichkeiten für spezielle Bevölkerungsgruppen (z. B. SeniorInnen, MigrantInnen, spezielle Berufsgruppen, Menschen mit besonderen Bedürfnissen, etc.) neu zu entwickeln und anzupassen und diese Kurskonzepte wissenschaftlich zu evaluieren [10, 29]. Dafür ist eine enge Zusammenarbeit mit Vertretern dieser Gruppen notwendig, um diese Bevölkerungsgruppen besser zu erreichen und die Bedürfnisse und bisherigen Hindernisse im Zugang zu Reanimationstrainings herauszufinden.

Weitere Aufgaben für die Zukunft sind einerseits der stetige Ausbau eines österreichweiten First-Responder-Netzes, andererseits die (psychologische) Betreuung der First Responder nach und zwischen Einsätzen. Oft wird das Engagement dieser als selbstverständlich gesehen, und die möglichen psychologischen Folgen der belastenden Einsätze werden nicht bedacht. Dazu wurde bereits ein standardisiertes Unterstützungssystem für Ersthelfer („first responder support system“ [FRSS]) vorgeschlagen, das sicherstellen soll, dass Ersthelfer auf ihre psychologische Gesundheit achten und ihre Motivation durch Wertschätzung aufrechtgehalten wird [30]. Ein solches System sollte österreichweit aufgebaut werden.

## Limitationen

Da den Fragebogen nur jene Organisationen beantworten konnten, an welche er gesandt wurde, besteht die Gefahr, dass relevante Organisationen nicht identifiziert wurden. Durch die Miteinbeziehung von ExpertInnen aus ganz Österreich haben die Autoren jedoch versucht, eine möglichst vollständige Liste zu erstellen. Rettungsorganisationen in Österreich spielen durch die angebotenen Erste-Hilfe-Kurse eine wesentliche Rolle in der Schulung von Laien. Aufgrund der fehlenden Antworten von fünf Rettungsdienstorganisationen sind die entsprechenden Daten nicht vollständig. Bei der Erstellung der Landkarte ist es nicht gelungen, den genauen Wirkungsbereich der einzelnen Organisationen darzustellen, sondern nur den Hauptsitz der jeweiligen Organisation.

## Schlussfolgerung

Die Aufklärung der Öffentlichkeit über die Basisreanimation und ein niederschwelliges Trainingsangebot können die Bereitschaft von Laien verbessern, bei einem Herzstillstand die Rettungskette zu starten und lebensrettende Sofortmaßnahmen einzuleiten. Die Politik sollte entsprechend tätige Organisationen unterstützen, um sowohl geografische Lücken schließen, als auch bestehende Strukturen weiter ausbauen zu können. Bei Kursen, in denen Reanimation gelehrt wird, muss ausreichend Zeit für das praktische Üben eingeplant werden. Neue Kurskonzepte für spezielle Bevölkerungsgruppen sollten entwickelt und evaluiert werden, und es sollte ein strukturiertes „Train-the-Trainer“-Konzept geben. Die Verwendung von gemeinsamen Ressourcen und Strukturen zwischen verschiedenen lokalen und nationalen Organisationen sollte angestrebt werden, um diese noch effektiver zu nutzen.

## Supplementary Information

Fragebogen
