# Supplementary material for: Layperson basic life support education in Austria: An overview
Source: Wien Klin Wochenschr. 2024 Feb 1;136(23-24):683–90. [Article in German] doi: 10.1007/s00508-024-02331-7 (PMC11631987; doi:10.1007/s00508-024-02331-7)
Supplement: Supplementary file 1 — Fragebogen [file 508_2024_2331_MOESM1_ESM.pdf]

Fragebogen

Österreichische Organisationen und Initiativen mit Wiederbelebungsbezug

Young Austrian Resuscitation Council (YARC)

Dieser Fragebogen wird vom Young Austrian Resuscitation Council (YARC) in Zusammenarbeit mit dem Österreichischen Rat für Wiederbelebung/Austrian Resuscitation Council (ARC) organisiert. Der Young ARC ist ein Teil des ARCs und hat unter anderem als Ziel junge Menschen, die sich für Reanimation und Notfallmedizin begeistern zu vernetzen. Ziel dieses Fragebogens ist die Erstellung einer aktuellen Bestandsaufnahme von Organisationen und Initiativen, die in Österreich Laienreanimation für die Öffentlichkeit anbieten. Dabei wollen wir sowohl lokale, als auch österreichweit tätige Organisationen und Initiativen abdecken. Die Auswertung soll anschließend öffentlich auf der Website des ARC (www.arc.or.at) zur Verfügung stehen bzw. auch in einem deutschsprachigen Journal als kurze Übersicht veröffentlicht werden. Anhand dieser könnten einerseits geographische Lücken aufgedeckt- und andererseits Kooperationen und ein Erfahrungsaustausch von Organisationen und Initiativen mit denselben Zielen gefördert werden (bspw. durch den ARC). So kann erstmals eine entsprechende österreichweite Plattform gebildet werden.
Bearbeitungsdauer: ca. 25 Minuten

Natürlich werden nur Informationen veröffentlicht, die von den jeweiligen Initiativen (bzw. Organisationen) in diesem Fragebogen freigegeben werden (siehe Disclaimer). Fragen, die nicht veröffentlicht werden sollen, sollen bitte einfach nicht beantwortet werden. Falls keine Rückmeldung zum Fragebogen kommt oder keine Fragen ausgefüllt werden, wird die entsprechende Initiative / Organisation auf der zu erstellenden „Landkarte“ als zwar vorhanden, aber mit dem Label „keine Information verfügbar“ gekennzeichnet.
Disclaimer: Ich willige hiermit im Namen der Organisation bzw. der Initiative ______________________ ein, dass meine Antworten auf die Fragen dieses Fragebogens vom ARC sowohl in eigenen Medien, als auch im Rahmen einer wissenschaftlichen Publikation, veröffentlicht werden dürfen. Der Organisation bzw. der Initiative erwachsen daraus keinerlei Rechte. Eine Adaptierung der Antworten durch den ARC, die über eine Formatierung oder Rechtschreib- /Grammatikkorrektur hinausgeht, ist nicht zulässig.

____________ ______________________ ______________________

Ort/Datum Name Unterschrift

Es wird im Fragebogen aus Lesbarkeitsgründen ausschließlich der Begriff „Organisation“ verwendet, es ist hier jegliche Form einer Gruppierung (Initiative, Verein,…) gemeint.

- **Daten, Aufbau und Struktur der Organisation**
    - Name der Organisation?

      ____

    - Wann wurde die Organisation gegründet?

      ____

- Wo wurde die Organisation gegründet?

  ____

- Wo ist der jetzige Hauptsitz der Organisation?

  ____

- Gibt es weitere Standorte neben dem Hauptsitz innerhalb von Österreich?

  O Ja → Wo? _________

  O Nein

- In welchem Wirkungsbereich ist Ihre Organisation tätig?

  O am Standort / Hauptsitz

  O im Umkreis von 25 km

  O im Umkreis von 50 km

  O im Umkreis von 100 km

  O im gesamten Bundesland

  O über das Bundesland des Standorts hinaus → Wo? _____________

- Gibt es weitere Standorte außerhalb von Österreich?

  O Ja → Wo? _________

  O Nein

- Was waren die Ziele bei der Gründung?

  ____

- Waren das Lehren der Laienreanimation und die Awarenessbildung für die Laienreanimation und den plötzlichen Herztod das Hauptziel bei der Gründung?

  O Ja

  O Nein

- Welche der folgenden Punkte gehören zum aktuellen Leistungsbereich?

  O öffentliche Reanimationstrainings für Laien

  O Reanimationstrainings für Laien, aber in geschlossenem Kursformat

  O ERC-Kurse

  O Erste Hilfe Kurs Führerschein

  O Erste Hilfe Kurs allgemein Erwachsene

  O Erste Hilfe Kurs allgemein Kinder / Säuglinge

  O Erste Hilfe Kurs gemischt

  O Aufklärung, Awarenessbildung und Präventionsarbeit für Laienreanimation und den plötzlichen Herztod

  O Reanimationstrainings für medizinisches Fachpersonal

  O Rettungsdienst

O Weitere: ____________________________

- Wie ist die Struktur der Organisation aufgebaut?

  O Verein

  O Unternehmen

  O Andere: ______________________________

- Arbeitet die Organisation gewinnorientiert?

  O Ja

  O Nein

- Über welche verschiedenen Möglichkeiten finanziert sich die Organisation?

  O Kurskosten

  O Mitgliederbeiträge

  O Spendenkampagnen

  O Spenden bei öffentlichen Veranstaltungen

  O Förderungen von Gemeinden, Ländern und/oder Bund

  O Stiftung

  O Weitere: ____________________________

- Wie viele Mitglieder hat die Organisation derzeit?

  ____

- Hat die Organisation hauptamtliches Personal angestellt?

  O Ja, wenn ja wie viele? ______________

  O Nein

- Kann man sich bei der Organisation ehrenamtlich engagieren?

  O Ja, wenn ja wie viele Ehrenamtliche sind derzeit aktiv? ______________

  O Nein

- Wie viele Stunden leistet das ehrenamtliche Personal ca. pro Jahr?

  ____

- Welches Eigentum besitzt die Organisation betreffend die Laienreanimation?

  O Puppen

  O Übungsdefibrillatoren

  O Gebäude inkl. Kursräume

  O Fahrzeug, um Material zu Kursen zu bringen

  O Material wird ausgeliehen

  O Andere: _______________________________________

- **Kurse und Kursformate**
  - Für welche der folgenden verschiedenen Zielgruppen bietet die Organisation Reanimations- oder Erste Hilfe Kurse an?

    O Erste Hilfe Kurse für Firmen

    O Erste Hilfe Kurse für den Führerschein

    O Erste Hilfe Kurs für Kindernotfälle

    O Reanimationskurs für Kinder (z.B.: in Schulen)

    O Erste Hilfe Auffrischungskurse

    O Reanimationstrainings für Polizei

    O Erste Hilfe Kurse im Rahmen einer Ausbildung (z.B.: OP Assistenz)

    O Reanimationstrainings BLS oder ALS für Gesundheitsberufe

    O Reanimationstrainings für Laien im Zuge von öffentlichen Veranstaltungen

    O Reanimationstrainings für Laien im Zuge von terminisierten Kursen

    O Reanimationstrainings für MigrantInnen

    O Reanimationstrainings für speziellere Zielgruppen, z.B. Menschen mit besonderen Bedürfnissen

    O Weitere: ____

  - Gibt es ein festes Kurskonzept?

    O Ja

    O Nein

    O Nur für bestimmte Kursformate (Welche? _________________________________)

  - Bietet die Organisation Reanimationskurse kostenlos an?

    O Ja

    O Nein, Selbstkostenbeitrag

    O Nein, gewinnorientiert

    O Teils / teils

  - Werden die Kurse beworben?

    O Ja

    O Nein

  - Falls ja, wo werden die Kurse beworben?

    O Social Media

    O Lokale Zeitungen

    O Flyer

    O Plakate

    O eMail an Mitglieder

    O Weitere: ____

- **Reanimationskurse für erwachsene Laien:**
  In diesem Abschnitt werden Fragen zu reinen Reanimationskursen für Erwachsene Laien gestellt. Falls Sie diese nicht anbieten, dann beantworten Sie bitte nur die erste Frage und danach können Sie direkt zum nächsten Abschnitt gehen (Erste-Hilfe-Kurse)

  - In welcher Frequenz werden Reanimationskurse für Laien angeboten?
    O gar nicht (dann die Fragen in diesem Abschnitt freilassen)
    O Wöchentlich
    O Monatlich
    O einmal im Quartal
    O Halbjährlich
    O Jährlich
    O Unregelmäßig
    O Kurs nur auf Anfrage
    O Andere: ____

  - Wie lange dauert ein Reanimationskurs?

    ____

  - Wie viel Zeit ist innerhalb des Reanimationskurses für das praktische Üben der Reanimation vorgesehen?

    ____

  - Wo finden die Reanimationskurse statt?
    O Räumlichkeiten der Organisation
    O Bei Firmen
    O In Schulen
    O In anderen Ausbildungsstätten (z.B.: Polizeiakademie, Ausbildung für OP Assistenz)
    O Öffentliche Gebäude

  - Welche Qualifikation oder Ausbildung haben die KursleiterInnen der Reanimationskurse?
    O Erste Hilfe Lehrbeauftragter
    O ERC Instruktor
    O Medizinisches Fachpersonal
    O keine besondere Qualifikation erforderlich
    O Interne Schulung (Details:____)
    O Andere: ____

  - Sind die KursleiterInnen für die Reanimationskurse ehrenamtlich oder angestellt?
    O Ehrenamtliches Personal
    O Hauptamtliches Personal

O Beides

- Wird die Qualität des Reanimationskurs evaluiert?
  O Ja
  O Nein

- Falls ja, wie findet die Evaluation des Reanimationskurses statt?
  O Fragebögen
  O Test vor und nach dem Kurs für Überprüfung des Fortschritts
  O Prüfung
  O Weitere: ____

- Werden die KursleiterInnen des Reanimationskurses evaluiert?
  O Ja
  O Nein

- Falls ja, wie findet die Evaluation der KursleiterInnen der Reanimationskurse statt?
  O Regelmäßige freiwillige Weiterbildungen
  O Regelmäßige verpflichtenden Weiterbildungen
  O Prüfungen
  O Supervision durch Andere
  O Weitere: ____

- **Erste-Hilfe Kurse:**
  In diesem Abschnitt werden Fragen zu Erste-Hilfe-Kurse für Erwachsene gestellt. Falls Sie diese nicht anbieten, dann beantworten Sie bitte nur die erste Frage und danach können Sie direkt zum nächsten Abschnitt gehen (Reanimationskurse für Kinder)

  - In welcher Frequenz werden Erste Hilfe Kurse angeboten?
    O gar nicht (dann die Fragen in diesem Abschnitt freilassen)
    O Wöchentlich
    O Monatlich
    O einmal im Quartal
    O Halbjährlich
    O Jährlich
    O Unregelmäßig
    O Kurs nur auf Anfrage
    O Andere: ____

  - Wie lange dauert ein Erste Hilfe Kurs?

    ____

- Wie viel Zeit ist innerhalb des Erste-Hilfe-Kurses für das praktische Üben der Reanimation vorgesehen?

  ____

- Wo finden die Ersten Hilfe Kurse statt?
  O Räumlichkeiten der Organisation
  O Bei Firmen
  O In Schulen
  O In anderen Ausbildungsstätten (z.B.: Polizeiakademie, Ausbildung für OP Assistenz)
  O Öffentliche Gebäude

- Welche Qualifikation oder Ausbildung haben die KursleiterInnen für die Erste Hilfe Kurse?
  O Erste Hilfe Lehrbeauftragter
  O ERC Instruktor
  O Medizinisches Fachpersonal
  O keine besondere Qualifikation erforderlich
  O Interne Schulung (Details:_______________________________________)
  O Andere: ____

- Sind die KursleiterInnen für die Ersten Hilfe Kurse ehrenamtlich oder angestellt?
  O Ehrenamtliches Personal
  O Hauptamtliches Personal
  O Beides

- Wird die Qualität des Erste Hilfe Kurses evaluiert?
  O Ja
  O Nein

- Falls ja, wie findet die Evaluation für die Erste Hilfe Kurse statt?
  O Fragebögen
  O Test vor und nach dem Kurs für Überprüfung des Fortschritts
  O Prüfung
  O Weitere: ____

- Werden die KursleiterInnen der Ersten Hilfe Kurse evaluiert?
  O Ja
  O Nein

- Falls ja, wie findet die Evaluation für die KursleiterInnen der Ersten Hilfe Kurse statt?
  O Regelmäßige freiwillige Weiterbildungen
  O Regelmäßige verpflichtenden Weiterbildungen
  O Prüfungen

O Supervision durch Andere
O Weitere: ____

- **Reanimationskurse für Kinder (Kinder werden unterrichtet):**
  In diesem Abschnitt werden Fragen zu Reanimationskursen für Kinder gestellt. Dieser Abschnitt soll Kurse abbilden, die speziell für **Kinder und Jugendliche als Teilnehmer** stattfinden. Falls Sie diese nicht anbieten, dann beantworten Sie bitte nur die erste Frage und danach können Sie direkt zum nächsten Abschnitt gehen (Reanimationskurse bei Veranstaltungen oder im öffentlichen Raum)

  - In welcher Frequenz werden Reanimationskurse für Kinder angeboten?
    O gar nicht (dann die Fragen in diesem Abschnitt freilassen)
    O Wöchentlich
    O Monatlich
    O einmal im Quartal
    O Halbjährlich
    O Jährlich
    O Unregelmäßig
    O Kurs nur auf Anfrage
    O Andere: ____

  - Wie lange dauert ein Reanimationskurs für Kinder?

    ____

  - Wie viel Zeit ist innerhalb des Reanimationskurses für das praktische Üben der Reanimation vorgesehen?

    ____

  - Wo finden die Reanimationskurse für Kinder statt?
    O Räumlichkeiten des Vereins oder der Organisation
    O Bei Firmen
    O In Schulen
    O In anderen Ausbildungsstätten (z.B.: Polizeiakademie, Ausbildung für OP Assistenz)
    O Öffentliche Gebäude
    O Öffentlichen Veranstaltungen

  - Welche Qualifikation oder Ausbildung haben die KursleiterInnen der Reanimationskurse für Kinder?
    O Erste Hilfe Lehrbeauftragter
    O ERC Instruktor
    O Medizinisches Fachpersonal
    O keine besondere Qualifikation erforderlich
    O Interne Schulung (Details:____)

O Andere: ____

- Sind die KursleiterInnen für die Reanimationskurse für die Kinder ehrenamtlich oder angestellt?

  O Ehrenamtliches Personal

  O Hauptamtliches Personal

  O Beides

- Wird die Qualität des Reanimationskurs für die Kinder evaluiert?

  O Ja

  O Nein

- Falls ja, wie findet die Evaluation des Reanimationskurses für die Kinder statt?

  O Fragebögen

  O Test vor und nach dem Kurs für Überprüfung des Fortschritts

  O Prüfung

  O Weitere: ____

- Werden die KursleiterInnen des Reanimationskurses für Kinder evaluiert?

  O Ja

  O Nein

- Falls ja, wie findet die Evaluation der KursleiterInnen der Reanimationskurse für Kinder statt?

  O Regelmäßige freiwillige Weiterbildungen

  O Regelmäßige verpflichtenden Weiterbildungen

  O Prüfungen

  O Supervision durch Andere

  O Weitere: ____

- **Reanimationskurse bei Veranstaltungen oder im öffentlichen Raum:**
  In diesem Abschnitt werden Fragen zu Reanimationskurse bei Veranstaltungen oder im öffentlichen Raum gestellt. Falls Sie diese nicht anbieten, dann beantworten Sie bitte nur die erste Frage und danach können Sie direkt zum nächsten Abschnitt gehen (Über Kurse hinausgehende Inhalte / Tätigkeiten).

  - In welcher Frequenz werden Reanimationskurse bei Veranstaltungen oder im öffentlichen Raum angeboten?

    O gar nicht (dann die Fragen in diesem Abschnitt freilassen)

    O Wöchentlich

    O Monatlich

    O einmal im Quartal

    O Halbjährlich

    O Jährlich

    O Unregelmäßig

    O Kurs nur auf Anfrage

O Andere: ____

- Wie lange wird pro TeilnehmerIn geplant?

  ____

- Wie viel Zeit ist innerhalb des Reanimationskurses für das praktische Üben vorgesehen?

  ____

- Wo finden die Reanimationstrainings statt?
  O Öffentlichen Veranstaltungen
  O Öffentlicher Raum im Rahmen von Aktionstagen mit Wiederbelebungsbezug (z.B.: World Restart a Heart)
  O Räumlichkeiten der Organisation
  O Öffentliche Gebäude

- Welche Qualifikation oder Ausbildung haben die InstruktorInnen?
  O Erste Hilfe Lehrbeauftragter
  O ERC Instruktor
  O Medizinisches Fachpersonal
  O keine besondere Qualifikation erforderlich
  O Interne Schulung (Details:____)
  O Andere: ____

- Sind die InstruktorInnen für die Reanimationskurse ehrenamtlich oder angestellt?
  O Ehrenamtliches Personal
  O Hauptamtliches Personal
  O Beides

- Wird die Qualität des Reanimationskurs bei Veranstaltungen oder im öffentlichen Raum evaluiert?
  O Ja
  O Nein

- Falls ja, wie findet die Evaluation des Reanimationskurses bei Veranstaltungen oder im öffentlichen Raum statt?
  O Fragebögen
  O Test vor und nach dem Kurs für Überprüfung des Fortschritts
  O Prüfung
  O Weitere: ____

- Werden die InstruktorInnen des Reanimationskurses bei Veranstaltungen oder im öffentlichen Raum evaluiert?
  O Ja
  O Nein

- Falls ja, wie findet die Evaluation der InstruktorInnen des Reanimationskurses bei Veranstaltungen oder im öffentlichen Raum statt?
  O Regelmäßige freiwillige Weiterbildungen
  O Regelmäßige verpflichtenden Weiterbildungen
  O Prüfungen
  O Supervision durch Andere
  O Weitere: ____

- **Über Kurse hinausgehende Inhalte / Tätigkeiten:**

Dieser Abschnitt dient der Abfrage von Tätigkeiten und Projekten mit Bezug zur Laienreanimation, welche jedoch das klassische Reanimationstraining oder Erste-Hilfe Kurse überschreiten. Falls Ihre Organisation in diesem Bereich nichts anbietet, können Sie diesen Abschnitt überspringen.

- Führen Sie Awarenessbildung bzgl. Laienreanimation durch?
  O Ja
  O Nein

- Falls ja, wie?
  O Social Media
  O Öffentlichen Veranstaltungen
  O Teilnahme an Aktionstagen mit Wiederbelebungsbezug (z.B.: World Restart a Heart)
  O Interviews in Fernsehen
  O Initiviativenforderungen/Vorschläge an die Politik
  O Weitere: ____

- Hatten Sie in der Vergangenheit Werbekampagnen, welche auf die **Laienreanimation** aufmerksam machen sollten?
  O Ja
  O Nein

- Falls ja, in welchen Medien wurde die Kampagne geführt?
  O Facebook
  O Instagram
  O Twitter
  O TikTok
  O eMail Verteiler
  O Radio

O Fernsehen

O Zeitung

O Plakate

O Flyer

O Weitere: ____

- Hatten Sie in der Vergangenheit Werbekampagnen, welche auf den **plötzlichen Herztod** aufmerksam machen soll?

  O Ja

  O Nein

- Falls ja, in welchen Medien wurde die Kampagne geführt?

  O Facebook

  O Instagram

  O Twitter

  O TikTok

  O eMail Verteiler

  O Radio

  O Fernsehen

  O Zeitung

  O Plakate

  O Flyer

  O Weitere: ____

- Arbeitet die Organisation zurzeit in Kooperationen mit anderen Initiativen mit Wiederbelebungsbezug zusammen?

  O Ja (Welche? _____________________________)

  O Nein

- Ist die Organisation regelmäßig mit Ständen bei öffentlichen Veranstaltungen vertreten?

  O Ja (Welche? _____________________________)

  O Nein

- Hat die Organisation an Aktionstagen mit Wiederbelebungsbezug (z.B.: Restart a Heart day, internationaler Herztag, Woche der Wiederbelebung, etc.) teilgenommen?

  O Ja

  O Nein

- Falls ja, wie sah die Teilnahme aus?

  O Aufmerksam machen über Social Media

  O Kostenloses Reanimationstraining für Öffentlichkeit an dem Tag

  O Start einer Werbekampagne

  O Weitere: ____

- **Wissenschaftliche Begleitung der Projekte:**
  Dieser Abschnitt ist in zwei Teile unterteilt. Die ersten Fragen richten sich an Organisationen, bei welchen bisher bereits eine wissenschaftliche Mitarbeit / Begleitung stattgefunden hat und die letzten zwei Fragen an die, bei welchen bisher noch keine wissenschaftliche Mitarbeit stattgefunden hat. Bitte auch dementsprechend nur die zutreffenden Fragen beantworten.

  - Gibt es eine Kooperation mit einer medizinischen Universität?
    O Ja (Welche? ______________________________)
    O Nein

  - Führt die Organisation selbst Studien mit Bezug zur Laienreanimaton durch?
    O Ja
    O Nein

  - Falls ja, Links mit Publikationen:

    ____

  - Beteiligt sich die Organisation bei Studien mit Bezug zur Laienreanimaton, nimmt aber hier selbst dafür nicht die Leitungsfunktion ein?
    O Ja
    O Nein

  - Falls ja, Links mit Publikationen:

    ____

- Folgende 2 Fragen sind nur für Organisationen, bei welchen bisher noch keine wissenschaftliche Mitarbeit stattgefunden hat:

  - Was sind die Gründe, warum bisher keine wissenschaftliche Mitarbeit stattgefunden hat?
    O Zeitliche Valenzen
    O Finanzierung
    O Fehlender Kontakt zu wissenschaftlichem Partner
    O Zu wenig Vorerfahrungen beim wissenschaftlichen Arbeiten
    O Kein Interesse
    O Andere: ____

  - Besteht das Interesse an zukünftiger Mitarbeit bei wissenschaftlichen Projekten?
    O Ja
    O Nein

- **Zukunftspläne**
  - Welche Projekte planen Sie für Zukunft?

    ___

- **Feedback:**
  - Bitte geben Sie noch eine eMail-Adresse für mögliche Rückfragen an:

    ___

  - Wir würden uns freuen, wenn Sie uns ein kurzes Feedback über diesen Fragebogen geben können:

    ___
